# Surgical tracheostomy in a cohort of COVID-19 patients

**DOI:** 10.1007/s00106-021-01021-4

**Published:** 2021-03-05

**Authors:** Patrick J. Schuler, Jens Greve, Thomas K. Hoffmann, Janina Hahn, Felix Boehm, Bastian Bock, Johannes Reins, Ulrich Ehrmann, Eberhard Barth, Karl Traeger, Bettina Jungwirth, Martin Wepler

**Affiliations:** 1 Department of Oto-Rhino-Laryngology, Head and Neck Surgery, Ulm University Medical Center, Frauensteige 12, 89075 Ulm, Germany; 2 Department of Anesthesiology and Intensive Care, Ulm University Medical Center, Ulm, Germany; 3 Department of Anesthesiology and Intensive Care, Ehingen Hospital, Ehingen, Germany

**Keywords:** Tracheostomy, Coronavirus, Surgery, Mortality, Ventilation, Tracheostomie, Corona, Chirurgie, Sterblichkeit, Beatmung

## Abstract

**Background:**

One of the main symptoms of severe infection with the new coronavirus‑2 (SARS-CoV-2) is hypoxemic respiratory failure because of viral pneumonia with the need for mechanical ventilation. Prolonged mechanical ventilation may require a tracheostomy, but the increased risk for contamination is a matter of considerable debate.

**Objective:**

Evaluation of safety and effects of surgical tracheostomy on ventilation parameters and outcome in patients with COVID-19.

**Study design:**

Retrospective observational study between March 27 and May 18, 2020, in a single-center coronavirus disease-designated ICU at a tertiary care German hospital.

**Patients:**

Patients with COVID-19 were treated with open surgical tracheostomy due to severe hypoxemic respiratory failure requiring mechanical ventilation.

**Measurements:**

Clinical and ventilation data were obtained from medical records in a retrospective manner.

**Results:**

A total of 18 patients with confirmed SARS-CoV‑2 infection and surgical tracheostomy were analyzed. The age range was 42–87 years. All patients received open tracheostomy between 2–16 days after admission. Ventilation after tracheostomy was less invasive (reduction in PEAK and positive end-expiratory pressure [PEEP]) and lung compliance increased over time after tracheostomy. Also, sedative drugs could be reduced, and patients had a reduced need of norepinephrine to maintain hemodynamic stability. Six of 18 patients died. All surgical staff were equipped with N99-masks and facial shields or with powered air-purifying respirators (PAPR).

**Conclusion:**

Our data suggest that open surgical tracheostomy can be performed without severe complications in patients with COVID-19. Tracheostomy may reduce invasiveness of mechanical ventilation and the need for sedative drugs and norepinehprine. Recommendations for personal protective equipment (PPE) for surgical staff should be followed when PPE is available to avoid contamination of the personnel.

## Abstract (de)

**Hintergrund:**

Ein Hauptsymptom der schweren Infektion mit dem neuen Coronavirus‑2 (SARS-CoV-2) ist das hypoxämische Atemversagen aufgrund einer Viruspneumonie, welches eine mechanische Beatmung erfordert. Eine längere mechanische Beatmung erfordert ggf. eine Tracheostomie, welche aufgrund des erhöhten Kontaminationsrisikos jedoch umstritten ist.

**Ziel:**

Bewertung von Sicherheit und Auswirkungen der chirurgischen Tracheostomie auf Beatmungsparameter und Überleben bei Patienten mit COVID-19.

**Studiendesign:**

Retrospektive Beobachtungsstudie zwischen 27. März und 18. Mai 2020 auf einer COVID-Intensivstation in einem Krankenhaus der Tertiärversorgung.

**Patienten:**

Patienten mit COVID-19 wurden wegen schweren hypoxämischen Atemversagens mit offener chirurgischer Tracheostomie behandelt.

**Messungen:**

Klinische Daten und Beatmungsparameter wurden retrospektiv aus medizinischen Unterlagen entnommen.

**Ergebnisse:**

Analysiert wurden die Daten von 18 Patienten mit chirurgischer Tracheostomie bei bestätigter SARS-CoV-2-Infektion. Die Altersspanne betrug 42–87 Jahre. Bei allen Patienten erfolgte zwischen 2 und 16 Tage nach der Aufnahme eine offene Tracheostomie. Anschließend war die Beatmung weniger invasiv, die Lungen-Compliance nahm zu und die Sedierung konnte reduziert werden. Es starben 6 von 18 Patienten. Alle chirurgischen Mitarbeiter waren mit FFP3-Masken oder mit motorbetriebenen luftreinigenden Atemschutzmasken ausgestattet.

**Schlussfolgerung:**

Die vorliegenden Daten legen nahe, dass eine offene chirurgische Tracheostomie ohne schwerwiegende Komplikationen durchgeführt werden kann. Diese kann die Invasivität der mechanischen Beatmung sowie den Bedarf an Beruhigungsmitteln und Katecholaminen verringern. Falls verfügbar, sollten Empfehlungen für die persönliche Schutzausrüstung befolgt werden.

## Introduction

It is presumed that the transmission of the new coronavirus‑2 (SARS-CoV-2) from animal to human occurred at the Huanan Seafood Market in Wuhan in December 2019. Since then, millions of people globally have been infected with the virus. In most cases, the infection displays a mild or asymptomatic course, in a larger cohort in China described in up to 80% of patients [35]. However, other patients develop a severe course of corona virus disease (COVID-19) with viral pneumonia and acute respiratory failure, which was described to occur in 17% of patients in a larger cohort in Germany [16].

The risk factors for the development of severe infection are reported to be advanced age, male sex and metabolic syndrome, which includes hypertension, high body mass index (BMI) and diabetes [33, 36]. These patients frequently develop hypoxemia because of severe acute respiratory failure, which requires intensive care with mechanical ventilation [13]. Currently, the main challenge for medical systems around the world is the shortage of patient beds on intensive care units (ICU), together with ventilation machines and qualified staff [11, 19].

In general, tracheostomy is favored in patients with respiratory failure requiring prolonged mechanical ventilation because it has been reported to facilitate weaning from ventilation [3] and therefore potentially increase the availability of ICU beds [14, 18]. However, in COVID-19 patients, tracheostomy itself and the optimal time point is a matter of considerable debate, mainly because of the increased risk for contamination of the medical staff during the procedure [2, 21]. The American Academy of Otolaryngology–Head and Neck Surgery suggests delaying tracheostomy in these patients for as long as possible [9]. They recommend performing the procedure only in those patients who display clinical signs of improvement, which implies a reduced virus load. This is typically the case after 2–3 weeks of ventilation. Other recommendations are to perform tracheostomy not before two negative SARS-CoV2 tests have been obtained [10] or only when the expected chance of recovery is high [9]. Importantly, all front of neck airway (FONA) procedures in COVID-19 patients are potentially associated with increased aerosol generation and virus exposure. Therefore, special precautions for personal protection should be followed by the surgeons [4, 38].

In the COVID-19 patients at our department, tracheostomy was performed at the earliest convenience because it may facilitate the ventilation of these patients and improve patient recovery. We decided to perform an open surgical tracheostomy by our Ear, Nose and Throat (ENT) specialists in all patients because of potential advantages compared to a dilatational tracheostomy: potentially lower risk for contamination during the procedure due to surgical preparation of the trachea, secured airway also during accidental dislocation of the tracheal tube when placing patients into prone position [3], waiving of bronchoscopy with additional aerosol production [26], and obesity as a frequent comorbidity in COVID-19 patients as a relative contraindication for dilatational tracheostomy.

## Methods

### Study population.

All included patients (*n* = 18) had confirmed infection with SARS-CoV‑2 and needed mechanical ventilation because of severe hypoxemic respiratory failure (Horovitz index < 150 mm Hg [PaO_2_ {partial pressure of arterial oxygen in the systemic arterial blood, mm Hg} divided by F_i_O_2_ {fraction of inspired oxygen, %}]) [18]. In general, all patients on mechanical ventilation due to COVID-19 on our ICU were intended for tracheostomy. In 4 patients of our COVID-19 cohort during March to May 2020, we decided to not perform tracheostomy due to severe multiorgan failure (MOF) from the very beginning of the disease [23]. The study was approved by the local ethics committee (#124/20). Informed consent was waived, and only anonymized data were analyzed.

### Confirmation of SARS-CoV-2 infection.

All patients in the study tested positive for SARS-CoV‑2 infection by deep throat swabs (*n* = 7), by bronchial lavage (*n* = 5) or both (*n* = 6). Screening for virus RNA was performed by reverse transcription polymerase chain reaction (RT-PCR).

### Blood, ventilation, and hemodynamic parameters.

Parameters were determined on admission to ICU and daily during intensive care treatment. The following ventilation parameters were determined once daily during invasive ventilation: peak pressure, PaO_2_, F_i_O_2_, Horovitz index, positive end-expiratory pressure (PEEP), and lung compliance. In addition, the dose of sufentanil, midazolam and norepinephrine, as well as the need for neuromuscular blocking agents, transfusion or dialysis was documented daily. Patients were brought into prone position for 16 h daily if they showed severe hypoxemia under mechanical ventilation (Horovitz index < 150 mm Hg, [8]).

### Surgery.

Open tracheostomy in all study patients (*n* = 18) was performed in the patient’s ICU bed with sterile draping. In all patients, oral nutrition was stopped for a minimum of 6 h prior to surgery. All included patients (*n* = 18) received open surgical tracheostomy, for which they received neuromuscular blocking agents to avoid coughing or pressing. Each procedure was performed by two head and neck surgeons, two scrub nurses and two anesthetists. All involved staff were well experienced. Overall, six different ENT surgeons and six different anesthetists were involved, who were protected by N99-mask and facial shield (*n* = 6) or by powered air-purifying respirator (PAPR, *n* = 6). PAPR equipment was provided by 3M (Versaflo, St. Paul, MN, USA) and PM (e-breathe, Mönchengladbach, Germany). Scrub nurses used N99-masks with a facial shield. Surgical steps were performed using Björk’s technique [17]. Before surgical opening of the trachea, ventilation was stopped, and the cuff was deflated. The transoral tube was advanced well into the trachea to decrease aerosol generation and viral exposure due to possible cuff dysfunction caused by the surgical instruments. For advancing the tracheal cannula, ventilation was stopped again, and the oral tube was removed with deflated cuff. All patients were provided with a 9.0 CH high to low cuffed (34.0 mm) tracheostomy tube (Covidien^TM^, Mansfield, MA, USA, 9.0 mm inner diameter and 12.2 mm outer diameter). In one patient, leakage at the tracheostoma was detected immediately after the procedure, so that 9.0 CH tracheal tube was changed to a 10.0 CH high to low cuffed (35.0 mm) tracheostomy tube (Covidien^TM^, Mansfield, MA, USA, 10.0 mm inner diameter and 13.6 mm outer diameter). All other surgical procedures were uneventful.

### Statistical analysis.

Continuous and categorical variables were presented as median and interquartile range (IQR) or absolute range. Due to the retrospective observational study design and therefore the irregular number of patients at each day before and after tracheostomy, we waived any statistical analysis and present our data only descriptively.

## Results

### Clinical characteristics of the patients

From March 27 and May 18, 2020, open surgical tracheostomy was performed on COVID-19 patients (*n* = 18) because of acute respiratory failure, but without severe MOF at a tertiary care university hospital in Germany. The age range was 42–87 years, with most of the patients being male (15/18, 83.3%). Patients’ baseline characteristics, including pre-existing conditions, are presented in Table 1.

**Table 1:** Baseline characteristics

	Survivor	Nonsurvivor
*Patients (n)*	12 (67%)	6 (33%)
*Age, median (range)*	63 (42–81)	75 (58–87)
*Sex (male/female)*	10/2	5/1
*BMI* ^a^*, median (range)*	32 (25–39)	28 (26–37)
*Patients on extracorporeal membrane oxygenation (ECMO)*	2 (17%)	0 (0%)
**Pre-existing conditions**		
Hypertension	9 (75%)	4 (67%)
Obesity (BMI > 30)	8 (67%)	2 (33%)
Diabetes	6 (50%)	2 (33%)
Coronary artery disease	3 (25%)	1 (17%)
Hyperlipoproteinemia	6 (50%)	1 (17%)
Chronic kidney disease	2 (17%)	2 (33%)
Chronic obstructive pulmonary disease	3 (25%)	0 (0%)
Obstructive sleep apnea syndrome	2 (17%)	0 (0%)
Hypothyroidism	3 (25%)	0 (0%)
Cancer ^b^	2 (17%)	1 (17%)
**Diagnostics** ^c^** (*n*)**		
Chest X‑ray	14	4
Pulmonary CT scan	7	3
Throat swap positive	10	3
Bronchial lavage positive	9	2
*Days between oral intubation and surgical tracheostomy (median; range)*	10 (2–16)	7 (4–12)

^a^ Body mass index (BMI) is body weight in kilograms divided by the square of the height in meters

^b^ Cancer included bladder cancer, testicular cancer, and acute myeloid leukemia

^c^ Patients may have received both chest X‑ray and pulmonary computed tomography (CT) scan or deep throat swab and bronchial lavage

### Surgical staff

No COVID-19 infection was detected in any of the staff who were involved in the surgical procedures. In addition, none of the staff had developed symptoms within three weeks after the procedure. All involved surgeons, anesthetists and scrub nurses were confirmed to be negative for SARS-CoV‑2 by one deep throat swab at the time of the data lock.

### Outcomes

**Fig. 1:**
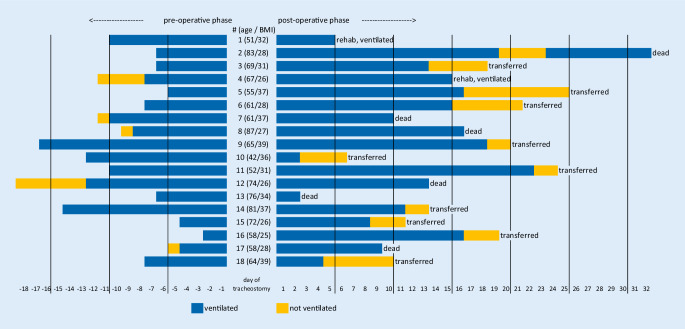
Outcomes for individual patients. Day of surgical tracheostomy is defined as day 0. Preoperative phase includes day −18 until day −1. Postoperative phase includes days 1 to 32. *Rehab* early rehabilitation facility. *BMI* body mass index in the body weight in kilograms divided by the square of the height in meters. At the time of the data lock (May 18, 2020), 12 patients (66.6%) had been discharged from the ICU, and 6 patients (33.3%) had died

At the time of the data lock on May 18, 12/18 (66.6%) patients had been transferred to a peripheral care ward or rehabilitation center. In 5/18 (27.7%) patients, the tracheal cannula had been removed and the tracheostoma was covered before discharge (median 9 days after tracheostomy). However, 2/18 (11%) patients were transferred to a rehabilitation center under invasive ventilation via a tracheal tube. In total, 10/18 (55.5%) patients had been taken off ventilation, and 6/18 (33.3%) patients had deceased (Fig. 1).

### Ventilation and hemodynamic parameters

**Fig. 2:**
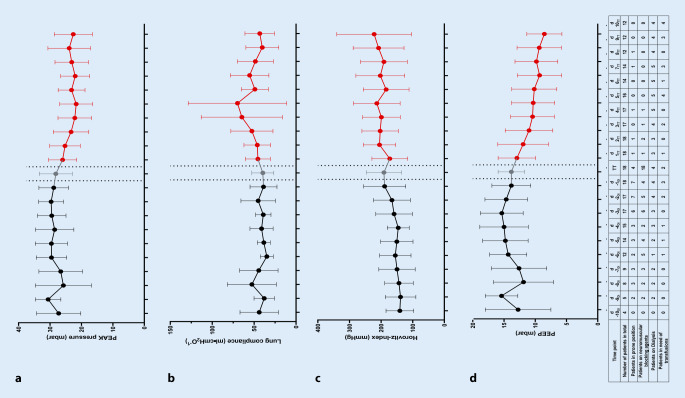
Ventilation parameters in the period from 10 days before surgical tracheostomy until 10 days after tracheostomy: **a** peak pressure (mbar), **b** static lung compliance (tidal volume divided by driving pressure, ml · cmH_2_O), **c** Horovitz index as a marker of oxygenation (partial pressure of arterial oxygen divided by the fraction of inspired oxygen, mm Hg), and **d** positive end-expiratory pressure (PEEP, mbar). After surgery, the number of patients in need for prone position and neuromuscular blocking agents was reduced whereas the number of patients requiring dialysis and blood transfusion increased (table). Data are presented as median and interquartile range

Peak pressure could be slightly reduced over time after tracheostomy. Lung compliance increased over time at least during the first five days after tracheostomy (Fig. 2a, b). The Horovitz index as a marker for oxygenation slightly increased over time after tracheostomy, whereas the PEEP could be reduced. The course of Horovitz index and PEEP is displayed in Fig. 2c, d. The number of patients in prone position was reduced from seven on the day before tracheostomy to one patient at the day after tracheostomy. The number of patients requiring neuromuscular blocking agents was reduced from four to one. The maximum number of patients requiring dialysis or blood transfusion was five and four, respectively (Fig. 2).

#### Sedative medication.

**Fig. 3:**
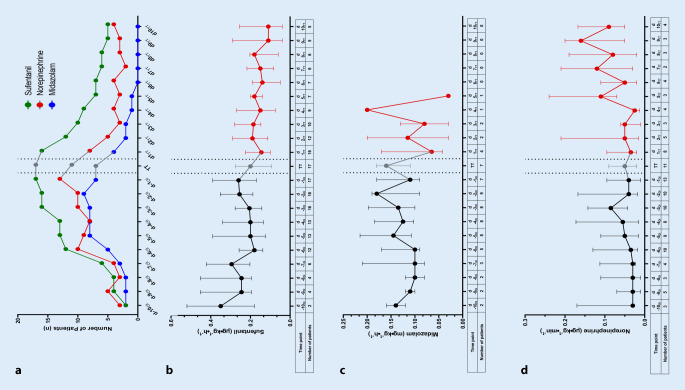
Sedative medication*.* **a** Number of patients receiving midazolam, sufentanil, or norepinephrine at the different time points during ventilation. **b** Doses of intravenous sufentanil (μg · kg^−1^ · h^−1^) and total number of patients receiving sufentanil (table). **c** Doses of intravenous midazolam (μg · kg^−1^ · h^−1^) and total number of patients receiving midazolam (table) at the different time points, and **d** doses of intravenous norepinephrine (μg · kg^−1^ · h^−1^) and total number of patients receiving norepinephrine (table) at the different time points. Sedation was managed according to the Richmond Agitation and Sedation Scale (RASS). Data are presented as median and interquartile range

After tracheostomy, the number of patients receiving sufentanil and midazolam decreased (Fig. 3a). In line with a reduction in sedation, the number of patients in need for norepinephrine to maintain hemodynamic stability decreased as well.

### Infection parameters

On admission, the median of the infection parameters C‑reactive protein (CRP), interleukin (IL)-6, brain-type natriuretic peptide (BNP) and procalcitonin were above the normal range in the peripheral blood. Leukocytes were in the normal range and lymphocytes were decreased (Table 2).

**Table 2:** Laboratory results on admission to ICU

Data point; median (range)	Survivor (*n* = 12)	Nonsurvivor (*n* = 6)	Norm range
Urea (mmoL/L)	11.3 (3.3–21.6)	10.8 (4.1–14.1)	3–9.2
Creatinine (µmoL/L)	105 (65–196)	139 (55–201)	59–104
Glomerular filtration rate (CKD-EPI)	53 (33–104)	37 (25–109)	–
Bilirubin (µmoL/L)	10 (6–45)	11 (4–19)	2–21
Aspartate aminotransferase (U/L)	82 (53–126)	52 (24–128)	< 50
Alanine aminotransferase (U/L)	39 (22–134)	33 (12–65)	< 45
Gamma-glutamyl transferase (U/L)	74 (14–821)	30 (24–75)	< 60
Alkaline phosphatase (U/L)	70 (29–345)	51 (36–77)	40–130
Creatine kinase (U/L)	434 (58–1741)	218 (10–2841)	20–200
Myoglobin (µg/L)	269 (43–1191)	161 (21–740)	28–72
Troponin (ng/L)	28 (5–128)	46 (13–254)	< 14
Procalcitonin (µg/L)	1.35 (0.08–5.54)	0.33 (0.19–22.7)	< 0.046
D‑dimer (mg/L)	2.4 (1.0–5.1)	2.5 (1.7–3.9)	< 0.8
Ferritin (µg/L)	1,153 (812–3717)	1,356 (280–8453)	4–665
C‑reactive protein (mg/L)	200 (52–437)	63 (2–115)	< 5
Lactate dehydrogenase (U/L)	349 (219–591)	377 (180–697)	< 250
BNP (pg/mL)	813 (116–3869)	2,766 (236–9670)	< 486
Interleukin‑6 (pg/mL)	246 (30–375)	447 (88–3140)	< 7
White blood cell count (×10^9^/L)	9.1 (3.0–19.0)	5.8 (3.9–11.1)	4.4–11.3
Lymphocyte count (×10^9^/L)	0.8 (0.4–3.7)	0.5 (0.3–0.7)	1.2–3.5
Platelet count (×10^9^/L)	207 (6–682)	116 (53–204)	150–450

*BNP* Brain-type natriuretic peptide, *ICU* intensive care unit

## Discussion

This single-center retrospective and observational study describes the clinical course of 18 critically ill COVID-19 patients with acute respiratory failure requiring mechanical ventilation. We included all patients with confirmed SARS-CoV‑2 infection, who received surgical tracheostomy because of invasive mechanical ventilation from March 27 to May 18, 2020.

Tracheostomy is a common procedure in critically ill patients who require prolonged mechanical ventilation and cannot be extubated. In COVID-19 patients, the optimal time point for tracheostomy is matter of considerable debate, mainly because of the high risk for virus transmission during the procedure [2, 21, 38]. The American Academy of Otolaryngology–Head and Neck Surgery suggests delaying tracheostomy in patients with COVID-19 for as long as possible [9]. They recommend performing the procedure only in those patients who display clinical signs of improvement, which implies a reduced virus load. This is typically the case after 2–3 weeks of ventilation [23]. Although delaying tracheostomy for patients with COVID-19 might reduce infectious risks for staff, extended duration of oral intubation would include continuation of sedation as well as mechanical ventilation. Prolonged sedation increases the risk for critical illness myo- and neuropathy, which would lead to a prolonged ICU stay [12, 23]. A tracheostomy can facilitate weaning from ventilation through a reduction in sedation with a faster conversion into a spontaneous ventilation mode and therefore potentially increase the availability of ICU beds, which may be important especially during the COVID-19 pandemic [23]. General recommendations in non-COVID patients with prolonged mechanical ventilation are to perform tracheostomy around day ten [32]. Early tracheostomy is frequently defined in the time frame of one week after intubation [30]. Overall, decision making for tracheostomy in COVID-19 patients include considerations as reduced risk for ventilator-associated respiratory muscle atrophy, ability to communicate, cumulative effects of a reduced sedation, and maintenance of ICU capacity for an early time point as well as potential risks to health-care workers due to virus contamination [23] and to patients due to a potential risk of increased mortality for later time points beyond day 14–21 [15, 20, 28].

However, tracheostomy in general may facilitate some beneficial effects in COVID-19 patients and potentially be performed earlier considering the clinical presentation of the patient [22]:

I. In our institution, patients were placed in prone position for 16 h per day when they displayed severe hypoxemia under mechanical ventilation (Horovitz index < 100 mm Hg). Because most of our COVID-19 patients presented with an increased BMI, turning such patients is cumbersome and is associated with an increased risk for dislocation of the ventilation tube. This is particularly the case at times when ICU are understaffed and at the limit of their capacity. In patients with a surgical tracheostoma, the risk for cannula dislocation or accidental decannulation is reduced [3], and in case of a dislocation while patients are in prone position, re-cannulation is straightforward. Furthermore, in our cohort of COVID-19 patients, tracheostomy apparently reduced the number of patients in need for prone positing (Fig. 2).
II. In our patients, tracheostomy led to a slightly less invasive ventilation. Apparently, there was a reduction in PEEP and PEAK pressure together with a slight increase in lung compliance (Fig. 2). A decrease in PEEP as well as PEAK to ensure adequate oxygenation (PaO_2_ > 60 mm Hg) and ventilation (PaCO_2_ within normal range for the individual patient considering pH values within 7.35 and 7.45), respectively, are signs of clinical improvement of lung function. Some guidelines recommend postponing tracheostomy until the patient has a PEEP requirement of 10 cmH_2_O or less [1] so that patients more likely overcome the procedure, which stresses the value of PEEP to present a marker of lung function. Although there is a bigger range after tracheostomy, Horovitz index slightly increased after tracheostomy in our patient cohort, which indicates an improvement of oxygenation and lung function as well. A lower Horovitz index may present an independent risk factor for mortality in patients with COVID-19 [37].
III. SARS-CoV‑2 has been reported to be found in the brain and cerebral fluid of human COVID-19 patients [34]. However, COVID-19 has not been described to impair the human brain function to a great extent. Patients are typically fully awake until transoral intubation, and their need for sedative medication is particularly high to tolerate the transoral ventilation tube and invasive ventilation with a high peak pressure. However, an extended period of deep sedation is associated with a prolonged weaning time in case of clinical improvement. In our patients, who had received tracheostomy, sedative medication was promptly reduced and stopped when tolerated by the patients.
IV. As in every other patient with severe hypoxemic respiratory failure requiring mechanical ventilation, patients with COVID-19 are at risk for extubation failure with the need for re-intubation. In case of extubation, COVID-19 patients are left with a hyperresponsive bronchopulmonal system because of the viral infection. Furthermore, other institutions report and increased number of laryngeal edema after extubation in COVID-19 patients, often leading to stridor and the need for reintubation immediately. In many cases, these reintubations are reported to be difficult due to significant edema in patients who were originally straightforward intubations [5, 24]. An extubation failure may increase morbidity and mortality of COVID-19 patients even further [29] as well as increase the risk for health care workers due to aerosol generation. This is not the case in patients with tracheostoma because ventilation can be started and continued directly on the cannula via a closed loop system, independently from function of the upper airway.

The option to perform percutaneous dilatational tracheostomy had been discussed in our department because it is a less time-consuming procedure [3]. However, for the first pandemic period from March to May 2020 we decided against it, mainly because of the increased risk for virus transmission during pulling back of the dilator and opening of the trachea. Minor points were the increased risk for tube dislocation during the prone position and the fact that most of our patients presented with increased BMI and adipose stature, which is a relative contraindication for percutaneous dilatational tracheostomy. Also, other university hospitals in Germany perform a surgical tracheostomy due to a shorter and more controlled aerosol exposure as well as surgical control of bleeding and waiving of bronchoscopy as another aerosol generating procedure [26]. Indeed, the same arguments were used during the SARS pandemic in 2004 [6].

**Fig. 4:**
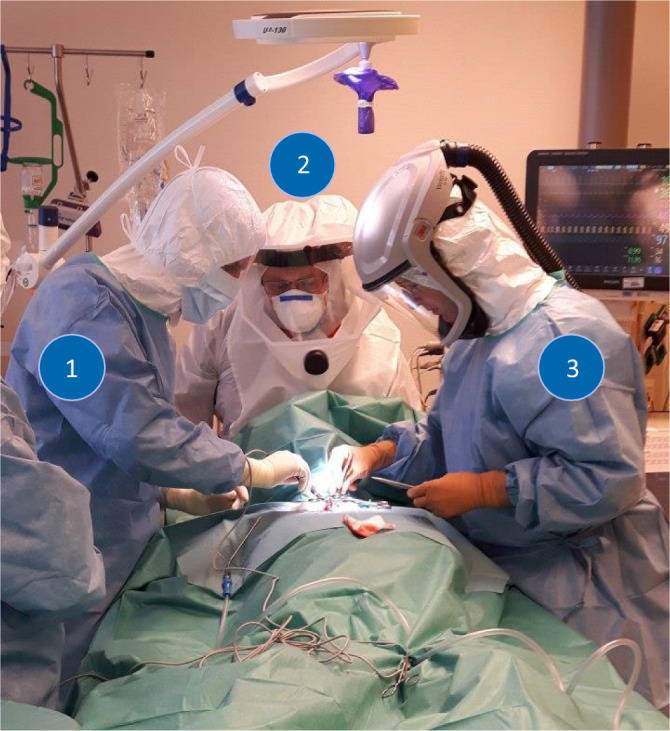
Surgery setup*. *Open surgical tracheostomy was performed in the patient’s bed in the intensive care unit. The patient was covered with sterile drapes. *1:* First surgeon on the patient’s right side with N99-mask and facial shield; *2:* anesthetist with powered air-purifying respirator (PAPR) at the patient’s head; *3:* second surgeon on the patient’s left side with PAPR. Reprint with permission © W. Schmidt, all rights reserved

All FONA procedures in COVID-19 patients are potentially associated with increased aerosol generation and virus exposure [38]. Most authors who suggest delaying tracheostomy in COVID-19 patients do so with reference to the increased risk for transmission during the procedure [9]. Fortunately, none of our staff, including surgeons, scrub nurses and anesthetists, were infected by SARS-CoV-2 at the time of data lock. Therefore, we suggest that surgical tracheostomy is a safe procedure in COVID-19 patients, when personal protection is worn and safety recommendations are followed [25]. In our setting, personal protection with N99-mask and face shield was equally effective as a PAPR (Fig. 4). Advantages of PAPR protection may include an increased safety by filtered air-flow and increased comfort. Furthermore, there are reports of SARS-CoV2 transmission despite protection via wearing a N99-mask during cardiopulmonary resuscitation [7]. However, with PAPR there is an increased risk for self-contamination when doffing as observed during the Ebola outbreak in 2014 [27]. Because the virus is characterized by a prolonged surface stability, special care must be taken when cleaning PAPR equipment [31].

### Limitations

Our study has several notable limitations. The number of patients treated is rather small. We found a series of apparent changes in ventilation parameters associated with the day of tracheostomy. We would like to underline that this association does not imply any correlation. The observed changes may as well have occurred during the intensive treatment period without any correlation to surgical tracheostomy.

## Conclusion

Our data suggest that surgical tracheostomy is a safe procedure in patients with COVID-19. Tracheostomy may support a positive course of disease in COVID-19-infected patients with severe hypoxemic respiratory failure requiring mechanical ventilation. Recommendations for personal protection of surgical staff should be followed when protective material is available. Overall, patient risk factors as well as disease severity together with local factors and expertise must be considered when decisions are made for tracheostomy and the specific procedure in patients with COVID-19.

## Declarations

For this article no studies with human participants or animals were performed by any of the authors. All studies performed were in accordance with the ethical standards indicated in each case.
